# Clinical Proteomics of Biofluids in Haematological Malignancies

**DOI:** 10.3390/ijms22158021

**Published:** 2021-07-27

**Authors:** Katie Dunphy, Kelly O’Mahoney, Paul Dowling, Peter O’Gorman, Despina Bazou

**Affiliations:** 1 Department of Biology, National University of Ireland, W23 F2K8 Maynooth, Ireland; katie.dunphy.2015@mumail.ie (K.D.); paul.dowling@mu.ie (P.D.); 2 Department of Haematology, Mater Misericordiae University Hospital, D07 WKW8 Dublin, Ireland; kelly.omahoney7@mail.dcu.ie (K.O.); pogorman@mirtireland.com (P.O.)

**Keywords:** biofluids, haematological malignancies, proteomics, biomarkers, leukaemia, lymphoma, multiple myeloma

## Abstract

Since the emergence of high-throughput proteomic techniques and advances in clinical technologies, there has been a steady rise in the number of cancer-associated diagnostic, prognostic, and predictive biomarkers being identified and translated into clinical use. The characterisation of biofluids has become a core objective for many proteomic researchers in order to detect disease-associated protein biomarkers in a minimally invasive manner. The proteomes of biofluids, including serum, saliva, cerebrospinal fluid, and urine, are highly dynamic with protein abundance fluctuating depending on the physiological and/or pathophysiological context. Improvements in mass-spectrometric technologies have facilitated the in-depth characterisation of biofluid proteomes which are now considered hosts of a wide array of clinically relevant biomarkers. Promising efforts are being made in the field of biomarker diagnostics for haematologic malignancies. Several serum and urine-based biomarkers such as free light chains, β-microglobulin, and lactate dehydrogenase are quantified as part of the clinical assessment of haematological malignancies. However, novel, minimally invasive proteomic markers are required to aid diagnosis and prognosis and to monitor therapeutic response and minimal residual disease. This review focuses on biofluids as a promising source of proteomic biomarkers in haematologic malignancies and a key component of future diagnostic, prognostic, and disease-monitoring applications.

## 1. Introduction

**Figure 1:**
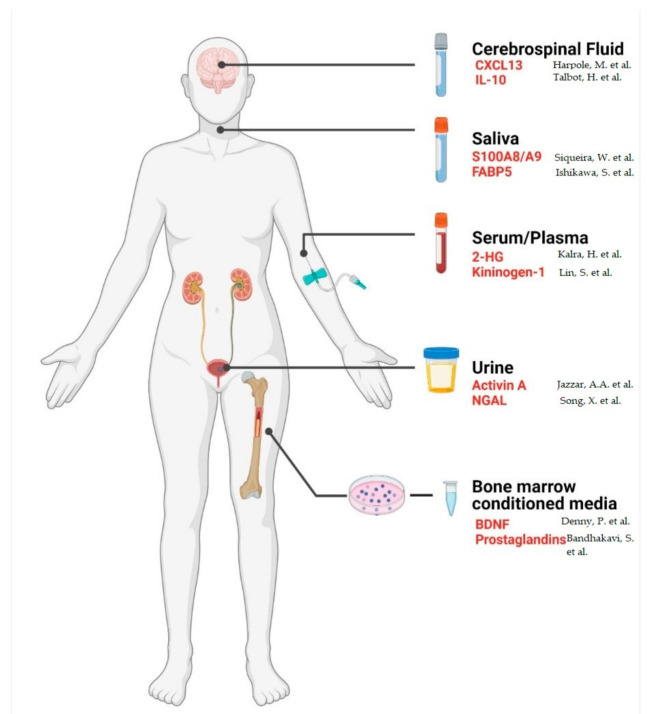
Biofluids are easily accessible and suitable for proteomic analysis in a clinical setting. Red font indicates promising protein biomarkers in haematological malignancies identified in the corresponding biofluid. CXCL13, C-X-C motif chemokine ligand 13; IL-10, interleukin 10; S100A8/A9, S100 calcium-binding protein A8/A9; FABP5, fatty acid binding protein 5; 2-HG, 2-hydroxyglutarate; NGAL, neutrophil gelatinase-associated lipocalin; BDNF, brain-derived neurotrophic factor. Created using BioRender.

Advances in proteomic technologies, protocols, and bioinformatic pipelines in recent decades have led to substantial progress in understanding the molecular phenotype of organisms by providing mechanistic insights into a wide range of cellular processes. Clinical proteomics aims to translate these discoveries to the clinic for the improvement of patient care. A major goal for many researchers in the biomedical community is the discovery of highly sensitive biomarkers to aid diagnosis, prognosis, and the monitoring of disease progression. Analysing changes in the proteome of physiologically or pathologically distinct samples (differential proteomics) enables researchers to identify proteins that are associated with different disease states [1]. Furthermore, the use of quantitative proteomic protocols, such as mass spectrometry-based techniques for discovery and targeted analyses, facilitates the quantitation of these proteins to identify candidate biomarkers with altered abundances for potential clinical applications [2]. Detecting and quantifying these protein markers in patient samples can contribute to an earlier diagnosis, a more accurate prognosis, and/or identifying therapeutic regimens that are more likely to benefit individual patients. Biofluids, such as serum, plasma, saliva, cerebrospinal fluid (CSF), urine, and bone marrow-conditioned media, are often considered reflections of a tumours’ molecular make-up, possessing genomic, transcriptomic, and proteomic indicators of disease (Figure 1). They represent a less invasive, less expensive, and more reproducible means of detecting disease-associated biomarkers when compared to invasive tissue biopsies (Table 1) [3].

Haematological malignancies are characterised as cancers that develop in the bone marrow, lymph nodes, and/or the blood from cells of the haematopoietic lineage. These malignancies include leukemias such as acute myeloid and chronic myeloid leukaemia, lymphomas such as Hodgkin’s lymphoma, and multiple myeloma (MM). The discouraging five-year survival rate, high rate of relapse, and incurability of certain blood cancer subtypes emphasises the need to identify novel therapeutic targets and biomarkers for the early detection of relapse and to assess disease progression following treatment.

## 2. Blood

Serum and plasma are often spoken about synonymously as they only differ from one another by the presence or absence of clotting agents [4]. Serum is retrieved following blood coagulation and centrifugation to remove fibrin clots, blood cells, and platelets. Plasma is prevented from clotting by adding an anti-coagulant, such as ethylenediaminetetraacetic acid (EDTA) or heparin, prior to extraction [5]. Despite the minor differences in composition, several studies have suggested that use of the incorrect sample source can lead to an improper diagnosis, hence why often either serum or plasma are preferred for certain assays [6,7,8]. Due to alterations in glucose levels between serum and plasma, serum is not recommended as a medium for the diagnosis of diabetes [9]. In addition, serum is the preferred sample source for quantitation of complement activation as EDTA-treated plasma must be transferred to veronal-buffered saline containing Ca^2+^ and Mg^2+^ to enable complement activation, and lepirudin as an EDTA replacement, prior to analysis [10]. 

### 2.1. Complexity of the Serum/Plasma Proteome

The high potential of serum/plasma as sources for protein biomarker discovery lies in their close proximity to all tissues, making their proteomic profiles reflective of the overall state of the organism [4,11]. An advantage of using serum or plasma for proteomic analysis is the minimally invasive, low-risk method of sample collection, which also facilitates sequential testing during the course of a disease. Despite the benefit of a high protein content, characterising the serum/plasma proteome can be challenging due to the nine-fold dynamic protein concentration range with just over 20 proteins including albumin, transferrin’s, immunoglobulins, and apolipoproteins, making up ≈99% of the serum/plasma proteome. The remaining 1% contains many low-abundant circulatory and secreted proteins that are often of more interest in research and as potential disease-associated biomarkers [12,13,14].

### 2.2. Methods for Analysing the Serum/Plasma Proteome

**Figure 2:**
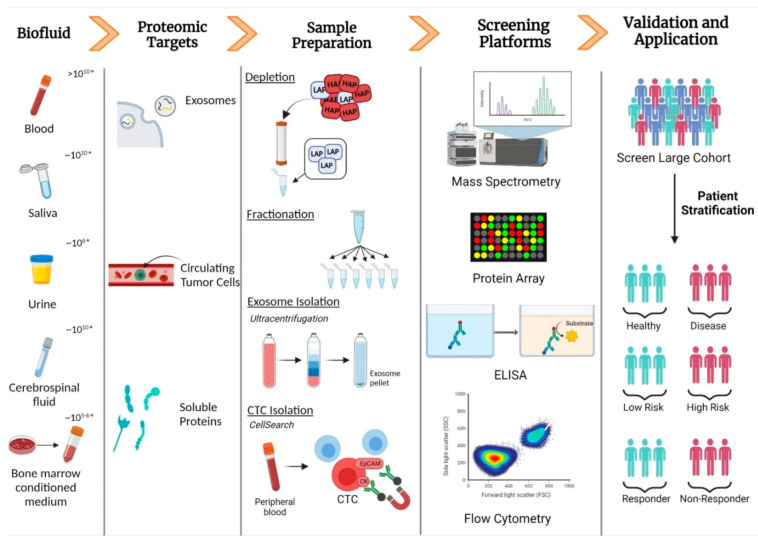
Schematic illustrating the steps involved in the detection of protein biomarkers in biofluids and their applications in a clinical setting. HAP, high-abundance protein; LAP, low-abundance protein; CTC, circulating tumour cell; ELISA, enzyme-linked immunosorbent assay; EpCAM, epithelial cell adhesion molecule; CK, cytokeratin. * Dynamic range of corresponding biofluid. Created using BioRender.

Several techniques have been employed to counteract the challenges of analysing such a dynamic and complex proteome. In order low abundance proteins to be effectively analysed, high-abundance protein (HAP) depletion, enrichment of specific low abundance proteins (LAPs), or fractionation to reduce sample complexity are often performed [15] (Figure 2). The removal of high-abundance proteins is necessary as they typically dominate the detection signals of analytical techniques, leaving low abundance proteins less likely to be detected and quantified [4,16]. Albumin is the most abundant protein (≈60% of total protein content) in plasma, making it a prime target for removal. Methods for albumin extraction include electrophoresis [17], dye-ligand chromatography [18], peptide affinity chromatography [19], and precipitation in organic solvents [20], as well as a recently developed molecularly imprinted polymeric hydrogel membrane (PHM) technique [21]. Another commonly used albumin depletion technique uses human serum albumin (HSA)-specific monoclonal antibodies to form an immunoaffinity resin, which successfully binds and removes HSA from the serum [22]. Nowadays, for the analysis of LAPs in serum/plasma, albumin is depleted in combination with other highly abundant proteins such as IgG, antitrypsin, IgA, transferrin, haptoglobin, fibrinogen, alpha2-macroglobulin, alpha1-acid glycoprotein, and apolipoprotein AI [15]. One of the most popular immunoaffinity kits, Multiple Affinity Removal Column Human 14 (MARS14), removes the 14 most abundant proteins from human plasma and has also been used for HAP depletion of other biological fluids [23]. Furthermore, combining ammonium sulfate precipitation and protein A affinity chromatography was found to be an affordable and effective method of plasma HAP removal [24]. A recent comparison of chemi-proteomic affinity-based probes against the popular MARS14 and ProteoMiner techniques revealed the affinity capture enrichment as an effective alternative to current methods with several advantages associated with the use of these probes [25]. Unfortunately, the discovery of biomarkers in serum/plasma can be obstructed during HAP depletion as protein–protein interactions between LAPs, such as cytokines, and off-target effects, can risk the concomitant removal of less abundant proteins that are of potential interest [26,27]. An investigation carried out by Chan et al. relied on a multi-dimensional peptide fractionation–tandem mass spectrometry method, instead of common HAP depletion methods to successfully identify low abundance proteins without their potential removal during the depletion process [11].

### 2.3. Detecting Biomarkers in Serum and Plasma

In addition to proteins, plasma, and serum are home to a diverse range of cells and other macromolecules including circulating tumour cells (CTCs), circulating tumour nucleic acids (ctNAs), and tumour-derived extracellular vesicles, namely, exosomes, which have been shed from tumours and their metastatic sites [28,29,30]. Detection of abnormal concentrations of these macromolecules in plasma/serum may lead to cancers being diagnosed at an earlier stage, facilitating a more accurate prognosis and improved chance of patient survival [3]. Other reviews on liquid biopsies have focused on the evaluation of circulating free nucleic acids in haematological malignancies [31].

CTCs are tumour cells that have been shed from the primary tumour site into the bloodstream, allowing malignant cells to travel to other parts of the body where they have the potential to metastasise [32]. Similar to circulating proteins, CTCs are generally rare in abundance in comparison to other cells in the blood with a ratio of about 1 CTC to approximately 10^9^ blood cells. In addition, their complexity and physiological variability can present complications for detection and analysis. As the number of CTCs present in the blood has been linked to cancer progression, response to therapy, and patient survival, many analytical techniques have focused on the enumeration of CTCs; however, the phenotypic characterisation of CTCs has lagged [3,33].

Techniques often used for the enrichment of CTCs from biofluids include density gradient centrifugation, microfluidic technologies, size-based separation, and immunoaffinity enrichment [34,35]. Immunoaffinity approaches of detecting CTCs in liquid biopsies include positive and negative selection in addition to immunomagnetic approaches involving substrate antibody immobilisation [3]. The immunoaffinity technique, *CellSearch*, is the only CTC detection and enumeration technique approved by the U.S. Food and Drug Administration (FDA) for use in the clinic. It detects CTCs of epithelial origin via the use of antibodies targeting epithelial cell adhesion molecule (EpCAM), cytokeratins, and CD45 (negative selection) in whole blood. Despite the ability of this technique to aid the prognosis of patient subgroups with solid cancers such as breast and colorectal cancer, drawbacks include the inability to detect mesenchymal-like CTCs and no possibility of further downstream analysis, which limits the molecular characterisation of the cells [29,34]. A recently described multifunctional platform mitigates the destructive nature of inductively coupled plasma mass spectrometry (ICP-MS) for CTC enumeration by incorporating aptamer-bound magnetic beads which facilitate CTC enumeration and the release of CTCs detected for further analysis. This combination of CTC enumeration and downstream molecular profiling represents a powerful technique for monitoring cancer progression and the personalisation of cancer treatments [36]. Inertial focusing using microfluidic devices is another label-free alternative that combats these issues via the size- and deformability-based separation of CTCs from whole blood [37]. Another promising in vivo method for CTC capture, the GILUPI CellCollector^®^, facilitates the screening of large volumes of blood using a medical wire coated with antibodies against EpCAM that is placed intravenously to capture CTCs that flow by [38]. Several studies have found CellCollector^®^ detects a larger number of CTCs than the CellSearch system; however, this technology has not yet been analysed for use in haematological malignancies [39,40]. Studies focusing on the proteomic analysis of the intracellular proteome of CTC cells are limited. A targeted single cell proteomics approach combining microfluidics-based enrichment of CTCs and Western blotting determined the biological expression levels of a panel of surface and intracellular proteins from single CTCs isolated from patients with estrogen receptor-positive (ER+) breast cancer [33]. Zhu et al. described a protocol for the proteomic profiling of CTCs comprising of immunodensity gradient enrichment of CTCs, laser capture microdissection, and finally ultrasensitive nano LC–MS/MS for protein identification and quantitation [41]. Additional separation technologies may be applied to isolate specific cellular populations in the serum or plasma for further proteomic analysis. For example, a Ficoll gradient is often used to isolate peripheral blood mononuclear cells which may contain the population of interest [42]. Microbeads conjugated to CD-antigens such as CD19 and CD34 are also used to obtain pure populations of target cells for further proteomic analysis [43].

Exosomes are lipid bilayer enclosed extracellular vesicles, approximately 30–100 nm in diameter, that are found in almost all biofluids including urine, saliva, blood, amniotic fluid, CSF, and conditioned media of cells [44]. Exosomes possess a range of macromolecules including lipids, nucleic acids, and proteins, which have been reported to reflect the molecular makeup of the cells from which they are derived [45]. They are considered mediators of intercellular communication via the transportation of cargo capable of inducing physiological or pathological changes from host to recipient cells [46]. The natural ability of exosomes to be released and taken up by cells, their molecular similarities with the host cell, and presence in biofluids has attracted attention in cancer therapeutics and diagnostics, with researchers exploring their application in diagnostics, drug delivery, and tumour immunotherapy [47,48,49]. 

The analysis of tumour-derived exosomes has led to the identification of novel biomarkers to aid diagnosis, prognosis, and therapeutic decision making in various cancers, with many clinically relevant biomarkers currently being evaluated in clinical trials [50,51]. Despite challenges associated with their small size and low density, methods successfully applied in exosome isolation include ultracentifugation, size exclusion chromatography, microfluidic technologies, immunoaffinity capture-based techniques, and microchip-based techniques such as the exosome total isolation chip (ExoTIC) [52,53,54,55,56]. However, these techniques are not 100% efficient, with the co-isolation of other extracellular vesicles, such as microvesicles and ectosomes, often occurring during exosomal preparation [57]. Furthermore, “exosomal isolates” from human plasma samples have been reported to be contaminated with high-abundance plasma proteins such as albumin, which can hinder downstream proteomic applications such as mass spectrometry-based analysis via the suppression of peptide ion signals derived from exosomal proteins, thus complicating the proteomic profiling of exosomes [58,59]. In order to translate exosome-based biomedical research into widespread clinically available diagnostic and drug delivery techniques, we require improvements and optimisation of isolation techniques to guarantee the purity of the sample.

### 2.4. Serum/Plasma Biomarkers in Haematological Malignancies

Human serum and plasma are widely used biofluid sources for proteomic analysis of haematological malignancies. The derivation of blood cancers from cells of the haematological system indicates serum and plasma as rich sources of blood cancer-associated biomarkers. Many blood-based protein biomarkers, such as lactate dehydrogenase and β2-microglobulin, have been identified in haematological malignancies (Table 2). However, they often serve as complementary markers of disease meaning invasive procedures such as bone marrow biopsies are required in addition to blood-based tests to confirm diagnosis, response to treatment, and relapse. Furthermore, with the approval of more targeted therapies for the treatment of blood cancers, such as venetoclax (BCL2 inhibitor), detecting specific molecular signatures to personalise therapeutic regimens is becoming increasingly important.

Many proteins have been found to be differentially expressed in the serum of patients with haematological malignancies compared to healthy controls. Recently, a study carried out by Chanukuppa et al. used a combination mass spectrometry, gel electrophoresis, and enzyme-linked immunosorbent assays (ELISAs) to identify and validate a panel of five serum proteins: haptoglobin; kininogen 1; transferrin; apolipoprotein A1; and the well-known MM marker, albumin, as potential diagnostic and prognostic biomarkers in MM [78]. Interestingly, a recent peptidomics study incorporating supervised neural network analyses identified a serum-based diagnostic MM model consisting of four peptides capable of distinguishing between MM disease states including healthy controls, newly diagnosed MM, and patients in complete remission, illustrating the potential of this model as a minimally invasive means of monitoring disease progression and treatment efficacy. The four peptides were found to be derived from dihydropyrimidinase-like 2, platelet factor 4, alpha-fetoprotein, and fibrinogen alpha [79]. A number of malignancies have been found to be associated with mutations in the gene encoding isocitrate dehydrogenase 1 (IDH1) and IDH2, including acute myeloid leukemia (AML) and myeloproliferative neoplasms [80,81,82]. The enzymes derived from these mutated genes have altered activity, producing 2-hydroxyglutarate (2-HG), an oncometabolite found to be increased in the serum of AML patients with IDH mutations and reduced following response to treatment. Monitoring serum 2-HG levels using liquid chromatography tandem mass spectrometry (LC–MS/MS) has been incorporated in various clinical trials to determine the efficacy of novel treatments in AML with IDH mutations [83]. The use of this technique has been reported to result in variable reference cut-off values due to the presence of two enantiomers. A recent study by Bories et al. used a chromatographic separation technique developed by Poinsignon et al. in order to establish individual reference values for each enantiomer to facilitate routine clinical use of serum 2-HG as a biomarker for disease monitoring in AML [84,85]. In acute lymphoblastic leukemia (ALL), various serum proteins have been identified in recent years as candidate biomarkers including S100A8, coagulation factor XIII subunit A, and a panel of 9 serum-derived glycoproteins [86,87,88]. Studies incorporating larger cohorts and clinically relevant workflows are required to bring “potential” and “candidate” serum biomarkers from benchtop to clinical use, a difficult task in certain cases due to low reproducibility, a lack of method standardisation, and difficulties translating the test used during discovery to a clinical-grade technology [89]. Studies focusing on the proteomic cargo of serum/plasma-derived extracellular vesicles in haematological malignancies have revealed interesting results, identifying proteins associated with drug resistance [90], survival, proliferation [91], and myelosuppression [92]. Blast-derived exosomes isolated from the sera of AML patients were found to contribute to immune suppression, in part by the inhibition of natural killer (NK) cell functions. Exosome-derived transforming growth factor-β1 contributes to NK cell suppression via a signalling cascade resulting in the downregulation of natural killer group 2 member D (NKG2D), a transmembrane receptor essential for the cytotoxicity of NK cells [93]. Incubation of neutralising antibodies targeting TGF-β with TGF-β+ AML exosomes followed by co-incubation with the NK cell line, NK-92, restored the cytotoxic activity of NK cells, illustrating exosome-derived TGF-β as a potential therapeutic target for the restoration of immune cell cytotoxicity in AML [94]. Efforts are being made by several research groups to improve current methods of CTC isolation and detection in MM. High numbers of circulating malignant plasma cells in the peripheral blood of the pre-malignant conditions, monoclonal gammopathy of un-determined significance (MGUS), and smouldering MM (SMM), as well as active MM, are associated with an increased likelihood of disease progression and a poor prognosis [95,96]. In addition to CTC enumeration, genomic and proteomic analysis of CTCs represents a unique opportunity for the molecular characterisation of these cells to guide personalised medicine by identifying biomarkers and therapeutic targets in a non-invasive, longitudinal manner [97]. 

Cancer relapse is of foremost concern due to the high rate of recurrence among patients with haematological malignancies. Monitoring minimal/measurable residual disease (MRD) with high sensitivity is essential for the early detection of relapse in patients. Improving the sensitivity of blood-based MRD testing has been the central goal for many researchers in recent years to facilitate longitudinal sampling without subjecting the patient to numerous invasive procedures. Currently, clonoSEQ is the only FDA-approved next-generation sequencing (NGS)-based assay to detect MRD in bone marrow samples from acute lymphoblastic leukaemia (ALL) and MM patients and in bone marrow samples and peripheral blood from patients with chronic lymphocytic leukaemia [98,99,100]. Several studies have used mass spectrometry-based methods to assess MRD by detecting clonotypic tryptic peptides derived from monoclonal immunoglobulins in the serum of MM patients, demonstrating a high sensitivity and the ability to detect clonal Igs in the serum of MM patients deemed to be MRD-negative by multiparameter flow cytometry (MFC) [101,102]. The increase in the use of MS-based techniques in the evaluation of M-proteins in plasma cell disorders (PCDs) led the international myeloma working group (IMWG) to provide recommendations on the use of MS in PCDs, encouraging further research on MS-based techniques as a means of testing MRD in the peripheral blood of MM patients [103].

## 3. Saliva

Saliva has previously been referred to as “the mirror of the body” due to the presence of a pool of biological markers that provide insight into the internal pathological state of an individual [104]. Whole saliva is composed of fluids secreted from the major and minor salivary glands that are derived from both local and systemic sources, demonstrating saliva as a promising medium for diagnosis of both oral and systemic conditions [105]. The potential for saliva as a diagnostic biomarker has accelerated because of the fast, inexpensive, and non-invasive method of collection as well as the vast abundance of proteins and genetic molecules it hosts. The major and minor salivary glands secrete an abundance of proteins belonging to classes such as the proline-rich proteins, α-amylases, defensins, mucins, salivary cystatins, and histatins [106,107]. Thousands of salivary proteins have been identified and quantified in a variety of diseases including oral cancer, head and neck squamous cell carcinoma, and Sjögren’s syndrome using mass spectrometry techniques [108,109,110,111,112]. It has been suggested that saliva contains approximately 40% of the potential protein biomarkers found in cancer, stroke, and cardiovascular disease [106]. 

### 3.1. Exploring the Saliva Proteome

The challenges involved in characterising this proteome are similar to those faced when characterising the serum/plasma proteome, the predominant challenge being the presence of HAPs such as albumin and amylase, which contribute to at least 60% of the human salivary proteome [113]. Major advancements in HAP depletion and fractionation techniques have facilitated advanced characterisation of the salivary proteome. In 2010, Bandhakavi et al. identified 2340 human salivary components with the aid of hexapeptide libraries (ProteoMiner) and 3D-fractionation of tryptic peptides using sequential preparative isoelectric focusing; strong cation-exchange chromatography; and capillary-reversed-phase HPLC prior to mass spectrometric analysis [114]. With improvements in proteomic techniques, a more recent study carried out by Grassl et al. identified a total of 5500 salivary proteins by combining fractionation and LC–MS/MS techniques [108]. In addition, different physiological conditions including time of day, age, and diet account for enhanced variability in the saliva proteome [115,116]. 

### 3.2. Proteomic Analysis of Human Saliva

Analysing the human saliva proteome to discover disease-associated biomarkers has become a desirable goal in the world of clinical research. In order to translate saliva into a clinically reliable medium for disease diagnosis, prognosis and monitoring, standardisation of protocols, and sample collection and analytical protocols, in addition to clinical validation of potential biomarkers, is required must be established [104,117,118]. 

From 2002, the National Institute of Dental and Craniofacial Research (NIDCR) offered funding to progress saliva-based biomarker discovery and develop reliable technologies such as microelectrochemical systems (MEMS) for saliva diagnostics, resulting in the publication of the salivary proteome in 2008 [119,120]. This database was developed to help decode disease pathogenesis and to observe the effects of medication on the structure, composition, and secretion of all salivary secretory proteins [120]. On the basis of this model of the salivary proteome, a panel of highly discriminatory salivary proteomic biomarkers for oral cancer detection have been identified with sensitivities and specificities at 89% [121]. 

The non-invasive nature of saliva makes it an ideal medium for point-of-care (PoC) testing, reducing the need for specialist sample collection protocols [122]. To establish a saliva-based test, simplified workflows incorporating pre-analytical sample processing steps must be developed. A recent study by Johannsen et al. aimed to reduce the complexity of saliva posed by its high viscosity and non-Newtonian nature using a fully automated, PoC test-compatible, magnet-beating device which significantly reduced the viscosity of saliva [123]. Another research group developed a new on-chip immunoassay based on microfluidic capillary flow assay (MCFA) for the highly sensitive detection and quantitation of salivary cortisol, a well-known diagnostic biomarker in various mental disorders [124]. Interestingly, a recent study illustrated the potential of conductive polymer spray ionisation mass spectrometry (CPSI-MS) and machine learning as a PoC test to detect dysregulated metabolites indicative of oral squamous cell carcinoma (OSCC) in saliva [125]. The development of these reliable, highly sensitive PoC tests holds promise for future saliva-based diagnostic, prognostic, and/or predictive tests for haematological malignancies.

### 3.3. Saliva Biomarkers in Haematological Malignancies

Despite biomarker discovery being largely focused on serum/plasma sources, salivaomics has grown as a field of study for the detection of novel biomarkers in recent years. Chen et al. evaluated 30 common leukaemia-associated fusion gene transcripts in leukemic and healthy saliva samples. The RNA fusion transcripts detected in the saliva of leukemic patients correlated with bone marrow analysis and remained stable when stored at room temperature, indicating a new, accurate, and non-invasive method for detecting leukemic cancer, especially in children [126]. It has also been noted that patients suffering from acute leukaemia present with oral symptoms such as pallor, gingivitis, and gingival enlargement. A study Månsson-Rahemtulla et al. assessed whole saliva stimulated by paraffin chewing to increase flow rate, from patients with AML and ALL who were undergoing chemotherapy, with the results showing that patients with leukaemia had significantly higher peroxidase and amylase activity as well as an increased abundance of salivary proteins in comparison to healthy control subjects [127]. Streckfus et al. observed pre-, peri-, and post-chemotherapy variations in the salivary protein profile of a patient with mantle cell lymphoma (MCL), suggesting the use of saliva for monitoring disease progression and treatment efficacy [128]. ELISA analysis of salivary immunoglobulins IgA, IgG, and IgM revealed significantly decreased levels in paediatric ALL patients which may result in the development and potentiation of oral lesions during chemotherapeutic treatment [129]. Sjogrens syndrome (SS) is an autoimmune disease that affects the exocrine glands of the body, including the salivary glands, and is associated with a significantly higher risk of developing lymphoma [130]. A recent study revealed that the levels of the pro-inflammatory heterodimer, S100A8/A9, in saliva could discriminate between healthy, SS, and mucosa-associated lymphoid tissue lymphoma (MALT-L), illustrating its potential as a salivary biomarker for the monitoring of SS progression [131]. Katz et al. identified potential biomarkers of MM-associated bone disease in the saliva of MM patients. Elevated levels of compounds that elicit oxidative stress known as advanced glycation end products (AGEs), were found in the saliva of MM patients with bone lesions [132]. Furthermore, Tierney and colleagues performed an MS-based analysis to identify changes in the salivary proteome of patients with the pre-malignant condition, monoclonal gammopathy of undetermined significance (MGUS), and patients with active MM. Increased levels of fatty acid binding protein 5 (FABP5) were found to correlate with disease progression, indicating its potential as a salivary biomarker for monitoring MGUS transformation to MM [133]. As is often the case, validation studies incorporating targeted approaches such as multiple reaction monitoring (MRM) and ELISAs, as well as a large cohort of patients, are lacking. Targeted clinical validation is needed to isolate salivary biomarkers with authentic clinical relevance from the large number of potential biomarkers identified during large-scale proteomic studies [134]. This is required to advance biomarkers through the process of biomarker development and validation for genuine consideration as protein biomarkers with clinical applications in haematological malignancies.

## 4. Bone Marrow Conditioned Media 

Bone marrow-derived cells include hematopoietic stem cells, mesenchymal stromal cells, and endothelial progenitor cells that secrete combinations of growth factors, cytokines, exosomes, and microvesicles to become what is known as the secretome or the conditioned media [135]. Conditioned media (CM) contains surface proteins that have been shed from the cell membrane, as well as intracellular proteins released through secretory pathways or extracellular vesicles. Secreted proteins (secretome) are said to be encoded by approximately 10% of the human genome and have been found to be principal components of biological processes such as cell growth, differentiation, and angiogenesis by regulating cell-to-cell and cell-to-extracellular matrix interactions [136]. Altered secretome expression associated with malignant transformation is an ongoing area of investigation for the discovery of novel cancer biomarkers. 

In vitro cell culture-based studies are commonly used for secretome analysis. Secretory proteins are released from the cultured cells into the conditioned media which is collected and analysed using proteomic techniques such as mass spectrometry and antibody arrays. With the availability of a wide range of blood cancer cell lines and co-culture models of bone marrow stromal cells and hematopoietic cells, bone marrow CM represents a promising non-invasive alternative to direct clinical specimen analysis for identifying novel candidate biomarkers for further validation. In addition, unlike the serum/plasma proteome, issues associated with high-abundance proteins can be avoided by using serum-free media during cell culture. However, a potential drawback of conditioned media as a source of biomarker discovery is that the in vitro cell culturing methods do not completely reflect the in vivo tumour environment [109].

Mesenchymal stem cells (MSCs), which are an important element of the bone marrow microenvironment, can be described as self-renewing, multipotent cells that have the capacity to differentiate into various cell types such as osteocytes, chondrocytes, and adipocytes. The MSC secretome has been found to be central to the interactions of MSCs with cells in their local environment [137]. A study evaluating the effects of the MSC secretome on the K562 leukemic cell line found that the MSC secretome alone had an anti-proliferative effect and had an additive cytotoxic effect on leukaemia cells when combined with doxorubicin [138]. Another study found prostaglandins (PGs), which are key components of the MSC secretome, to be progressively elevated in MSCs derived from Fanconi anaemia (FA) patients with myelodysplastic syndrome and AML. The increased PGs resulted in the upregulation of NR4A-WNT/β-catenin signalling in co-cultured CD34+ cells which attenuates anti-leukemic immunity [139]. In AML, MSC-derived secretory factors, including stanniocalcin 1, were found to contribute significantly to the suppression of haematopoietic stem and progenitor cells (HSPCs) [140]. The analysis of MSC-derived extracellular vesicles revealed their role in normal and malignant haematopoiesis, which has recently been reviewed by Batsali et al. [141]. Exosomes containing gene regulatory proteins released by apoptosis-resistant AML cells have been implicated in conferring apoptotic resistance to neighbouring cells including AML blasts with a low anti-apoptosis index [142]. Extracellular vesicles derived from Hodgkin’s lymphoma were found to alter the secretome of fibroblasts, resulting in a cancer-associated fibroblast phenotype [143]. In primary effusion lymphoma (PEL), proteomic analysis of PEL cell secretome identified a number of proteins associated with growth and the immune response, which may represent potential biomarker candidates [144]. In B chronic lymphocytic leukemia (B-CLL), a nurse-like cell secretory protein, brain-derived neurotrophic factor (BDNF), was found to support the survival of B-CLL cells [145]. These studies demonstrate the myriad of secretory factors from malignant blood cells and neighbouring cells which influence the pathobiology of haematological malignancies, illustrating cancer tissue–proximal fluids and conditioned media as potential sources of biomarker discovery and therapeutic target detection.

## 5. Urine

Urine is produced through the filtration of blood in the kidneys to remove unwanted waste and excess fluids. Urine contains a wide variety of proteins which, in part, reflect the plasma proteome as well as the kidney proteome, suggesting a promising source for the detection and quantitation of disease-associated biomarkers [146]. The analysis of urine presents several advantages, the main advantage being the ability to collect large volumes in a non-invasive manner. In addition, urinary proteins are less susceptible to proteolysis, and the complexity of urine proteome is considerably lower than the plasma proteome, which simplifies the detection of changes in protein abundance between samples. However, the dynamic protein concentration range spans a magnitude of 106, signifying the need to remove high-abundance proteins such as albumin in order to detect low-abundance proteins [147]. HAP depletion/enrichment techniques, such as MARS, have been applied for urinary protein analysis [148]. Furthermore, the composition of the urine proteome is impacted by physiological conditions such as diet, medications, and exercise [149]. Mass spectrometry and antibody-based protein arrays are often used for the analysis of urine. A study Zhao et al. incorporating different separation strategies, including one- and two-dimensional LC–MS/MS, as well as gel-eluted liquid fraction entrapment electrophoresis followed by two dimensional LC–MS/MS, identified 6085 urinary proteins which make up the Human Urinary Proteome Database [147]. Capillary electrophoresis coupled with mass spectrometry (CE–MS) is worth mentioning as a promising technique for the detection of biofluid-based biomarkers, especially urinary biomarkers, in haematological malignancies. CE–MS is orthogonal to liquid chromatography (LC)–MS, whereby analytes are separated based on their electromigratory properties [150]. A recent study using CE–MS identified a panel of 19 significant peptides to distinguish between patients with clinically significant and non-significant prostate cancer to aid therapeutic decision making [151]. Capillary zone electrophoresis (CZE) is often used to detect M-protein when screening for myeloma and other monoclonal gammopathies [152].

In haematological malignancies, the urinary proteome has been probed to detect diagnostic and prognostic biomarkers as well as markers of nephrotoxicity [99,153,154]. A well-establish method of assessment of MM or other plasma cell dyscrasias is the detection of paraproteins in serum or urine by protein electrophoresis or immunofixation [99]. Efforts have been made in recent years to develop novel methods of paraprotein detection in urine due to the limitations associated with conventional methods, including low sensitivity and highly laborious protocols [155]. Nanomaterials have become increasingly popular with a wide range of applications in the biomedical field. Long et al. recently developed a highly sensitive and specific novel method of paraprotein detection in MM using macroporous ordered silica foams (MOSF) for Bence Jones protein enrichment coupled with matrix-assisted laser desorption/ionisation time-of-flight mass spectrometry (MALDI-TOF MS) [155]. The association of paraproteinemia with other haematological malignancies such as primary cutaneous marginal zone lymphoma [156] indicates the potential clinical use of urinary paraprotein detection. In addition to serum, 2-HG was found to be elevated in the urine of IDH1/2-mutated AML patients compared to wild-type controls. An optimal threshold to predict the presence of IDH1/2 mutations was established to be above 16,650 ng/mL in urine. Although the sensitivity of measuring urinary 2-HG levels to predict IDH1/2 mutations was lower than serum (0.5600 and 0.8039, respectively), urinary 2-HG remains a promising non-invasive biomarker that warrants further investigation [157]. The treatment of paediatric acute lymphoblastic leukaemia (ALL) using cytostatics and irradiation are associated with nephrotoxicity and an increased risk of kidney damage. A recent study identified higher levels of two markers of tubular injury, urinary kidney injury molecule-1 (KIM-1) and neutrophil gelatinase-associated lipocalin (NGAL), in the urine of ALL survivors compared to healthy controls [153]. Urinary NGAL was also found to be elevated in MM patients with renal impairment compared to MM patients without renal impairment [158]. In addition, urinary activin A was reported to be significantly increased in newly diagnosed MM patients compared to patients with pre-malignancy plasma cell dyscrasia and healthy donors. Urinary activin A levels correlated with tubular injury in MM, indicating its potential as a biomarker of renal impairment [159]. As with saliva, urine represents an ideal medium for point-of-care testing. Microfluidic devices have been developed for urinary biomarker marker analysis which can be adapted to create a diagnostic platform for a variety of diseases [160]. These developments, as well as recent studies identifying candidate urinary protein biomarkers in gastric and prostate cancer, present urine as a promising medium for further proteomic analysis in blood cancers [161,162]. 

## 6. Cerebrospinal Fluid

Cerebrospinal fluid (CSF) refers to the liquid that surrounds the brain and spinal cord. A lumbar puncture, or spinal tap, is used to collect CSF from individuals for subsequent analysis. Although the collection of this biofluid is more invasive than the other biofluids discussed in this review, the gold standard for diagnosis of central nervous system (CNS) infiltration due to haematological malignancies remains the cytological analysis of CSF in order to identify neoplastic cells [163]. CNS invasion is a severe complication of haematological malignancies resulting in significant morbidity and mortality. The incidence of CNS involvement is relatively rare but differs depending on the blood cancer subtype. Around 1% of myeloma patients and between 3 and 5% of childhood leukaemia patients have CNS involvement [164,165]. Cytological assessment of CSF to detect CNS involvement suffers from low sensitivity due to contamination with peripheral blood cells and the presence of disease at undetectable levels [166]. Therefore, efforts have been made in recent years to identify novel biomarkers of haematologic malignancy in CSF.

Mikhael et al. recently evaluated a multiplexed panel of protein biomarkers in the serum and CSF for the assessment of CNS involvement in acute lymphoblastic leukaemia (ALL) patients. Alterations were seen in matrix metalloproteinase 9 (MMP-9), inducible protein 10 (IP-10), vascular cell adhesion molecule-1 (VCAM-1), and interferon-γ levels, although these changes were not statistically significant. However, a study incorporating a larger cohort and longitudinal sampling may reveal significant proteomic changes in the CSF of ALL patients analysed before and during CNS infiltration [167]. CSF-based biomarkers in childhood leukaemias have recently been reviewed [164]. A recent study revealed soluble interleukin-2 receptor (IL-2R) levels in CSF as a useful diagnostic indicators of CNS infiltration in haematological malignancies, with the combined analysis of soluble IL-2R and autotaxin improving detection sensitivity in patients with lymphoma [163]. In B-lineage cell ALL, high expression levels of cortactin were found to be associated with increased transendothelial migration and bone marrow relapse. Interestingly, 100% of CNS infiltrated leukemic cells isolated from CSF samples from B-ALL patients expressed cortactin, indicating a potential role of this protein in CNS infiltration. Further studies correlating cortactin expression levels in haematological malignancies with CNS infiltration may provide insight into the molecular mechanisms associated with CNS involvement [168]. In a study by Mo et al., CSF was collected from pre- and post-treatment (after achieving a complete response) B-ALL patients with CNS involvement. Following an MS-based analysis, 10 significantly altered protein expression profiles were identified between the two cohorts. One protein found to be decreased following a complete response to therapy was secreted protein, acidic, cysteine-rich (SPARC), a protein involved in cell adhesion and migration that has previously been implicated as a poor prognostic factor of AML [169,170]. Despite the relatively small sample size used in this study, SPARC represents a promising prognostic biomarker of CNS-involved ALL and potential therapeutic target that warrants furthers investigation. 

Primary central nervous system lymphoma (PCNSL) is a rare form of intracranial, extranodal, non-Hodgkin’s lymphoma for which the gold standard of diagnosis is an invasive, stereotactic biopsy [171]. A systematic review of diagnostic biomarkers of CNS lymphoma (CNSL) identified 18 markers including microRNAs, surface proteins, and intracellular proteins in CSF that are of diagnostic value [172]. This review provided researchers with a basis for future validation studies of these markers in order to translate these candidate markers into clinically relevant diagnostic biomarkers. CXCL13 and IL-10 were found to be excellent diagnostic markers of CNSL and PCNSL, respectively, demonstrating high sensitivities and specificities and their relevance for assessment for clinical use [173,174]. Another prospective study identified a proliferation inducing ligand (APRIL) alone, or in combination with B cell activation factor (BAFF) as reliable diagnostic biomarkers. In addition, APRIL levels were found to correlate with methotrexate (MTX)-based polychemotherapy and disease relapse, illustrating its potential as a marker of therapeutic response [171]. Belimumab, an anti-BAFF monoclonal antibody, recently FDA-approved for the treatment of lupus nephritis, has been reported to enhance the effectiveness of small molecule inhibitors, such as idelalisib and venetoclax, in the treatment of CLL. Belimumab, in combination with established chemotherapies, may represent a promising therapeutic regimen for the treatment of CNS-involved leukaemia [175].

As described, progress has been made in recent years to identify novel CSF-based biomarkers in CNS infiltrated haematological malignancies, especially leukaemias and lymphomas. However, more research is required to translate these findings into clinically relevant technologies such as multiplexed ELISAs; protein arrays; or the targeted MS-based approach, multiple-reaction monitoring mass spectrometry (MRM–MS). Finally, standardised CSF pre-analytical protocols must be developed to ensure external factors, such as sample collection or storage, do not alter the levels of biomarkers detected during testing. For example, several studies have aimed to establish standardised pre-analytical procedures for measuring CSF-based biomarkers of Alzheimer’s disease [176,177].

## 7. Conclusions

Despite the difficulties that proteomic analysis of biofluids present, the field has improved considerably in terms of sample collection, analytical techniques, and bioinformatic analysis. Researchers have confronted challenges, such as the presence of HAPs, by developing various workflows to maximise protein detection and quantitation in biofluids to identify large numbers of promising biomarkers. However, the detection of highly sensitive biomarkers with sequential validation in a large cohort using technologies applicable in the clinic is urgently required for the non-invasive diagnosis of disease and prediction of treatment response. Clear standards for data acquisition as well as reliable methods for data comparison must be developed to simplify and expedite the translation of candidate biomarkers into those used in clinical settings. Overcoming these limitations will undoubtedly lead to the application of biofluid-based proteomic biomarkers to improve the diagnostic, prognostic, and predictive power of current methods used in the clinical assessment of haematological malignancies.

## Figures and Tables

**Table 1:** Advantages and disadvantages of tissue and biofluid-based proteomics for the detection of blood cancer-associated biomarkers.

Tissue-Based Proteomics		Biofluid-Based Proteomics	
Advantages	Disadvantages	Advantages	Disadvantages
Direct analysis of proteins from site of disease	Invasive procedure	Non-invasive	Not in direct proximity to the site of disease
Facilitates the study of the bone marrow microenvironment	Localised sampling bias due to heterogeneity of the bone marrow microenvironment	Ease of longitudinal sampling	High abundance proteins can hamper detection
Gold standard for diagnostic and prognostic applications	High cost	Low cost	
	Bone marrow biopsies can be painful procedures	Reflective of disease state	

**Table 2:** Current clinically used protein biomarkers in haematological malignancies.

Biofluid	Protein	Type of Blood Cancer	Technology	Clinical Purpose	References
Serum	Monoclonal immunoglobulin (M-protein)	Multiple myeloma	Serum protein electrophoresis immunofixation electrophoresis	Diagnostic and monitoring disease	[60]
	Free light chains (Bence Jones proteins)	Multiple myeloma	Immunoturbidimetric and immunonephelometric assays	Diagnostic and monitoring of patients with light-chain disease.	[61]
	Βeta 2-microglobulin	Multiple myeloma	Nephelometry immunoturbidimetry	Prognostic	[62,63,64,65,66,67,68,69]
		Acute leukaemia			
		Chronic leukaemia			
		Hodgkin’s lymphoma			
		Non-Hodgkin’s lymphoma			
	Lactate dehydrogenase	Multiple myeloma	Enzyme kinetics assay	Prognostic	[70,71,72,73,74]
		Acute leukaemia			
		Chronic leukaemia			
		Hodgkin’s lymphoma			
		Non-Hodgkin’s lymphoma			
	Uric acid	Acute myeloid leukaemia	Colorimetric enzyme assay	Prognostic	[75]
Urine	Monoclonal immunoglobulin (M-protein)	Multiple myeloma	Protein electrophoresis Immunofixation electrophoresis	Diagnostic and monitoring of disease	[76]
	Free light chains (Bence Jones proteins)	Multiple myeloma	Immunofixation electrophoresis Immunoturbidimetry	Monitor disease progression and response to therapy	[76]
Cerebrospinal fluid	Βeta 2-microglobulin	Lymphoma	Nephelometry	Indicative of central nervous system (CNS) involvement	[77]
		Leukaemia			

## Data Availability

Not applicable.
